# Dental practitioners’ knowledge, management practices, and attitudes toward collaboration in the treatment of temporomandibular joint disorders: a mixed-methods study

**DOI:** 10.1186/s12875-024-02398-1

**Published:** 2024-04-26

**Authors:** Muhammad Taqi, Syed Jaffar Abbas Zaidi, Saad uddin Siddiqui, Babar Zia, Maria Khadija Siddiqui

**Affiliations:** 1 Department of Community Dentistry, Dow Dental College, Dow University of Health Sciences, Karachi, Sindh Pakistan; 2 Department of Oral Biology, Dow Dental College, Dow University of Health Sciences, Karachi, Sindh Pakistan; 3 Department of Oral Medicine, Dow Dental College, Dow University of Health Sciences, Karachi, Sindh Pakistan; 4 Department of Community Dentistry, Jinnah Medical & Dental College, Dow University of Health Sciences, Karachi, Sindh Pakistan

**Keywords:** Temporomandibular Joint disorders, Physical therapy modalities, Interprofessional relations, Quality of Healthcare

## Abstract

**Background:**

Temporomandibular joint disorders (TMDs) are a variety of conditions that affect different parts of the temporomandibular joints (TMJ) and can cause orofacial pain and functional impairment. This study aims to investigate dental practitioners’ knowledge and management of Temporomandibular Joint Disorders (TMDs), particularly their knowledge of the role physical therapy plays in TMD treatment.

**Methods:**

A mixed-methods approach was adopted to provide a comprehensive view of current knowledge, management practices, and attitudes toward collaboration among dental practitioners in treating TMD. Data were collected from a convenience sample of 335 dentists in Karachi using a detailed questionnaire to assess their knowledge of the role of physical therapy in the treatment of TMD. Twenty dentists were chosen for face-to-face, in-depth interviews to explore their experiences and challenges in managing TMDs based on their responses to the administered questionnaire.

**Results:**

The cumulative quantitative and qualitative findings of the study revealed a landscape marked by individualized approaches to referral practices and significant gaps in interdisciplinary collaboration. Most practitioners holding a bachelor’s degree predominantly used medication (65.2%) and cause-specific treatment (65.3%) for TMD treatment. Thematic analysis of clinical efficacy and practitioner challenges in managing TMD revealed significant issues faced by dental professionals.

**Conclusions:**

The study successfully validated a questionnaire to understand dental practitioners’ knowledge regarding physical therapy in TMD treatment. The study identified significant gaps in knowledge and a lack of collaboration between dentists and physiotherapists. The limited referral practices highlighted in the study, along with insights from dentist interviews, emphasize the need for improved interdisciplinary approaches to managing TMDs within dental practice.

## Background

Temporomandibular joint disorders (TMDs) encompass conditions that impact aspects of the temporomandibular joint (TMJ). These conditions can result in pain and functional limitations [1]. TMD is a long-term orofacial pain condition leading to challenges in work productivity, social interaction, and overall quality of life [2]. Studies suggest that TMD becomes more prevalent with age and is more commonly observed among women than men [3]. Research conducted on the adult population in Pakistan indicates a TMD prevalence ranging from 62 to 66% [4, 5]. Generally, 10% of the population experiences TMJ pain, with 3.6–7% seeking treatment for their symptoms [6, 7].

Various TMDs are recognized by the Diagnostic Criteria for Temporomandibular Disorders (DC/TMD). These include acute and chronic TMD, simple and complex, as well as factors associated with cognitive, psychosocial, and behavioural functioning [8]. In chronic cases of TMD, multidisciplinary treatment is especially critical [9]. TMD pain can be treated by dentists, physical therapists (PTs), speech pathologists, physicians, and psychologists. For patients with TMD, treatment options that are least invasive and most cost-effective are most appropriate while considering TMD-associated factors such as parafunctional habits, poor posture, widespread pain, poor sleep, and depression [10].

TMD pain can be effectively treated with physical therapy (PT) [2]. PT is one of the non-invasive treatments shown to help patients with TMD, along with behavioural therapy and occlusal appliances [11]. One of the most important contributions of physical therapists is identifying the musculoskeletal components that cause symptoms [12]. TMJ-related pain can be treated by PTs the same way that other joints of the body because they are part of the musculoskeletal system. There are a variety of modalities available in PT to treat TMD pain secondary to inflammation, masticatory muscle pain, TMJ hypo/hypermobility, disc displacement, bruxism, and fibrous adhesion [13].

Studies have shown that manual therapy, jaw exercise, and postural reduction can reduce pain in patients with TMD while improving their mobility and function [13, 14]. Furthermore, physiotherapists (PTs) treat TMJ through different electrotherapeutic modalities such as electrical stimulation [15], ultrasound [16], acupuncture [17], and laser therapy [18]. The aim of all these treatment modalities is to reduce pain inflammation and reestablish correct flexibility and muscular strength to improve mobility and function, which further promotes the healing of tissues.

The management of Temporomandibular Disorders (TMDs) has been significantly advanced through a multi-modal treatment approach, as indicated by recent findings [19]. This approach, which synergistically combines dental and physical therapy, has been shown to be more effective than dental treatment alone [20]. The integration of these two disciplines offers a more comprehensive strategy for the treatment of TMD, highlighting the importance of interdisciplinary collaboration. Despite the demonstrated efficacy of this combined approach, a considerable knowledge gap exists among dental professionals regarding the benefits of collaboration with physiotherapists, especially in the context of Karachi [21]. Systematic reviews have further corroborated the success of this integrative treatment method [13, 14], emphasizing the need for increased awareness and cooperation between dentists and physiotherapists to achieve optimal patient outcomes. This necessity is further highlighted by the findings of List et al., who identified a significant lack of awareness among dentists about the advantages of working with physiotherapists [22]. This situation is particularly pronounced within the context of Karachi, suggesting a localized, yet critical, deficiency in interdisciplinary awareness. While numerous studies have emphasized the complexity of TMD and the need for multidisciplinary treatment, there remains a notable disconnect in the practical application of these insights. This is particularly evident in Karachi, where dentists’ familiarity with and utilization of physiotherapy in TMD treatment appears limited. Addressing this knowledge gap is imperative for optimizing TMD treatment outcomes, necessitating focused educational and professional development initiatives to foster a more integrated approach to the management of TMDs.

The underutilization of multidisciplinary approaches in TMD management could be attributed to several factors. Studies suggest that the lack of standardized training and guidelines for dentists in the management of TMD may contribute to this issue. Furthermore, cultural and systemic barriers within healthcare systems can also influence the extent of interdisciplinary collaboration [21]. This is consistent with the findings in Karachi, where traditional practices and a lack of exposure to multidisciplinary approaches may limit the adoption of integrated treatment strategies.

Moreover, the potential benefits of physiotherapy in TMD management, as highlighted in the literature, are often under-recognized. Recent studies have demonstrated the efficacy of physiotherapeutic interventions such as manual therapy and exercise in reducing TMD symptoms [19, 20]. However, these modalities remain underutilized in many dental practices, as indicated by the limited referral rates to physiotherapists. This disconnect points to the need for educational programs aimed at increasing dentists’ awareness of the benefits of physiotherapy in TMD management. The complexity of TMD necessitates a detailed comprehension of its various contributing factors. This underlines the importance of considering psychological and behavioral factors in TMD patients. This holistic perspective is essential for effective treatment but is often missing in traditional dental education and practice. Our study, therefore, aims to bridge this knowledge gap by exploring the current practices and perspectives of Karachi’s dentists regarding the role of physical therapy in TMD treatment.

The research question of this study is “How do dental practitioners’ knowledge and referral practices regarding physical therapy influence the management of Temporomandibular Joint Disorders (TMDs) in Karachi, and what are their personal experiences and challenges in treating these disorders?” This research question is multifaceted, encompassing both quantitative and qualitative aspects.

Thus, our primary objective is to investigate how dental practitioners’ knowledge and referral practices regarding physical therapy influence the management of Temporomandibular Joint Disorders (TMDs) in Karachi, Pakistan, focusing on validating an assessment tool designed to evaluate their understanding of the role of physical therapy in treating TMDs. The secondary objectives of this study are as follows:

- Determining the extent to which dentists collaborate with physiotherapists for TMDs management through referral practices.
- Investigate the types of TMDs treated in dental clinics across Karachi.
- Exploring the rich experiences and unique challenges of dentists regarding temporomandibular disorders (TMDs) and the role of physical therapy in its management.

These objectives are contextually relevant as the study aims to provide valuable insights into dentists’ knowledge and practices in Karachi. By focusing on these objectives, we aim to contribute to the development of more integrated and effective approaches for managing TMD, ultimately enhancing patient care and outcomes in this context.

## Methods

### Study design

This research was conducted as a sequential mixed-method study, beginning with a quantitative phase followed by a qualitative phase. Our approach was designed to first gather broad, generalizable data through the quantitative survey and then explore these findings through qualitative in-depth interviews based on lived experiences of the participants. The study was conducted from March 2023 to April 2023.

### Sampling and participants

The first part of the study comprised the quantitative component that addressed the following research question : “How do dental practitioners’ knowledge and referral practices regarding physical therapy influence the management of Temporomandibular Joint Disorders (TMDs) in Karachi?” This part was addressed through the survey data collected from dentists in Karachi, focusing on their knowledge of physical therapy’s role in treating TMD and their referral practices. For this purpose, a convenience sampling method was employed. The study involved dentists practicing in both public and private clinics in Karachi who consented to participate. Dentists submitting incomplete data and those practicing outside Karachi were excluded.

A total of 335 dentists were approached for participation in the quantitative survey. The sample size was determined using Open Epi, with the assumption of a 68% awareness rate regarding the role of physiotherapists in treating TMD [23], a 95% confidence interval (CI), and a 5% margin of error. The study questionnaire for the quantitative component was adopted from a previous study [24]. The second part of this study comprised of qualitative interviews. The qualitative component involved in-depth interviews with a purposive sample of 20 dentists selected from the survey participants based on their responses and experiences.

### Study instrument

For this study, the questionnaire was divided into six sections with closed-ended questions. The first section includes participant consent, a cover letter describing the study’s goal, assuring participants of the anonymity of their responses, the protocol for answering the questions, demographic information, prior education, and the general question about dental practice. Section two includes an inquiry regarding the current status and location of dental practice. Section three contains questions about the prior knowledge of dentists regarding the role of physiotherapists in treating TMDs. Section four contains questions regarding the TMD patient population. The questions in Sect. 5 were about referral practices for patients with TMD. In Sect. 6, the participants were asked about the need to learn about the benefits of collaboration with physiotherapists.

### Pretest

Before launching the main study, a pilot test of the inventory was conducted with 36 participants who were not enrolled in the primary investigation. The pretest aimed to assess comprehension and determine the average time to complete the questionnaire. An expert health professional performed the content validity of the questionnaire, ensuring that the content was appropriate and relevant for the intended audience. Internal consistency was determined using Cronbach’s alpha. This assessment gauged the consistency between responses to the questions across all three domains (knowledge, TMD patient population, and referral practices) as shown in Table 1.

The Pearson’s correlation coefficient (𝑟) was computed between individual items and the aggregate scores of respondents. Items with correlation values exceeding the threshold of 0.32 were considered to demonstrate strong validity. This threshold was derived from the critical values table for Pearson correlation, which is instrumental in determining statistical significance as shown in Table 2. After analysing the pilot data and obtaining feedback, the final version of the questionnaire was drafted for the main study.

**Table 1:** Cronbach’s alpha values

Domains	Alpha value	Mean ± S.D
Knowledge question	0.67	9.82 ± 2.33
TMD Patient population	0.81	32.1 ± 5.26
Referral practices	0.65	2.07 ± 1.56

**Table 2:** Pearson correlation coefficients for questions using a degree of freedom two and Critical value (0.32)

Knowledge	Correlations	*P* value
Prior to this survey, to what extent were you aware that physical therapists can treat patients with TMD by reducing jaw movements and restoring masticatory muscle function?	0.815	0.001
Prior to this survey, to what extent were you aware that cervical spine pain may be involved as a cause of masticatory region pain?	0.742	0.001
Before this survey, to what extent were you aware that physical therapy can improve TMD symptoms with oral exercises, manual therapy, and postural reduction?	0.771	0.001
**TMD patient population** What type of TMD have you evaluated and treated?		
TMJ Disc Displacement	0.362	0.025
TMJ Degeneration	0.330	0.043
TMJ Hypermobility	0.453	0.004
TMJ Hypomobility	0.370	0.022
Myofascial pain	0.587	0.001
Parafunctional Habits	0.349	0.032
From the options below, what do you normally include in evaluating these patients?		
TMJ palpation	0.601	0.001
Masticatory muscle palpation	0.484	0.002
Jaw movements	0.747	0.001
Signs of parafunctional habits	0.599	0.001
Occlusion	0.581	0.001
Do any of your patients with TMD present with neck pain during evaluation?	0.635	0.001
Do any of your patients with TMD present with headaches during evaluation?	0.334	0.041
Do any of your patients with TMD present with poor posture during evaluation?	0.554	0.001
When treating patients with TMD, what methods do you normally use?		
Bite splints/occlusal guards	0.549	0.001
occlusion correction/braces	0.473	0.003
Prescription of medication	0.588	0.001
Cause-specific treatment	0.568	0.001
**Referral practices** If you had referred a patient with TMD to a physical therapist, what would be the reason for the referral?		
Neck pain	0.487	0.002
Postural alteration	0.728	0.001
Masticatory muscle tenderness	0.657	0.001
Headaches	0.737	0.001

### Data collection

In this study, a questionnaire was designed using Google Docs and the survey link was distributed to participants via WhatsApp and email. After the initial invitation, reminder emails and WhatsApp messages were sent to participants to encourage survey completion. The initial invitation and subsequent reminder emails informed participants that they could be contacted for an interview after submitting their responses. The survey was anonymous, and participants did not receive any compensation. Each participant could only complete the survey once, and their responses were kept confidential.

### Interview protocol

The qualitative part of the study addressed the research question: “What are their personal experiences and challenges in treating these disorders?” This aspect was explored through in-depth interviews with practicing dentists. Interviews were completed with 25 patients; however, data saturation in qualitative terms was reached with the first 20 patients. No additional categories or themes emerged from the subsequent five interviews. Consequently, the thematic analysis focused on the pre-established codes derived from the initial 20 participants. These dentists were selected to represent diverse experiences, knowledge levels, and collaboration practices. In-depth face-to-face interviews were then conducted with selected participants. The interviews were audio-recorded and transcribed for qualitative data analysis. Participants were asked to share their unique perspectives and rich experiences of treating TMD, their referral practices, and their collaborations in treating TMD through semi-structured interviews. A single investigator conducted each interview, which lasted 45 min to an hour. Interview questions were designed to explore the reasons behind dentists’ knowledge, referral practices, and experiences related to TMD and physical therapy. The interview guide consisted of the following interview questions:

- Can you share your experiences or insights in treating patients with TMD?
- What factors have influenced your knowledge and beliefs about physical therapy for TMD?
- Could you describe your experiences collaborating with physiotherapists or other colleagues in managing TMD patients?
- What factors influence your decision to refer a patient to a physiotherapist or colleague for TMD management?

### Study cohort progression and inquiry flow

**Fig. 1:**
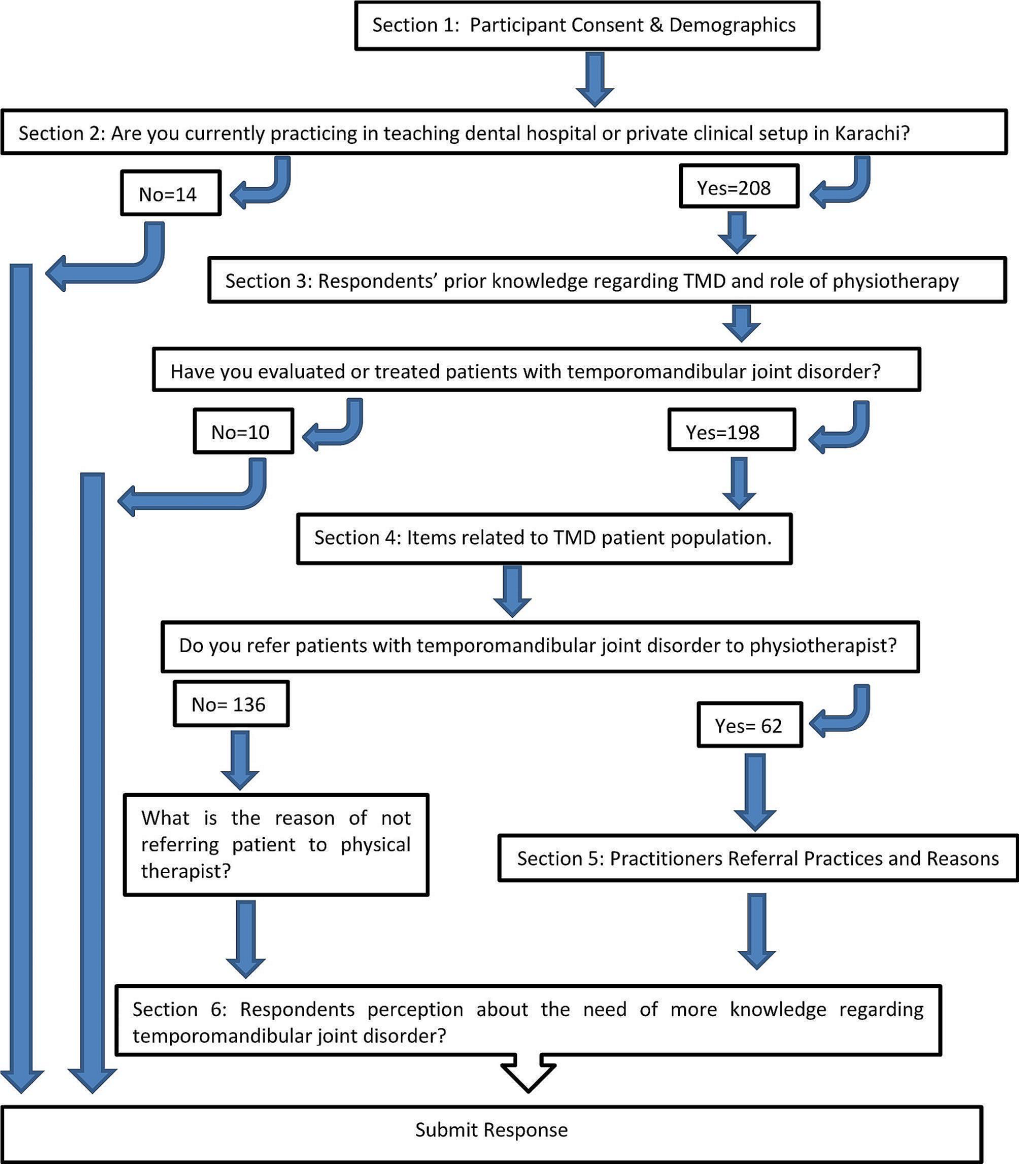
Study Flow Chart

In the design of our study, we employed the Skip Logic or Branching Logic feature offered by Google survey forms. This allowed us to create individualized paths within the survey, each tailored to a respondent’s prior answers. Our rationale for this approach was two-fold: Ensuring that participants only encountered questions relevant to their background and experience, thereby streamlining their survey experience. By presenting only pertinent questions, we aimed to minimize the potential for participants to respond out of mere obligation, a factor that could skew the data. To offer a clear depiction of how respondents navigated the survey based on their responses, a comprehensive flow chart was generated as shown in Fig. 1.

The investigation commenced with an initial pool of 222 practitioners. An early screening led to the exclusion of 14 practitioners who were not currently involved in dental practice and were directed to the submission stage. A further assessment excluded ten more practitioners, primarily due to their lack of experience evaluating or treating TMD patients. These individuals also proceeded to the form submission stage.

The heart of the study is encapsulated in Sect. 4, which probed into the practitioners’ referral habits concerning TMD of the participants. A total of 136 dentists indicated that they had never referred TMD patients to physiotherapists. This subset was further quizzed about their reasons for this non-referral pattern and then moved to Sect. 6.

On the other hand, a total of 62 practitioners confirmed their habit of referring TMD patients to physiotherapists. Their journey led them to Sect. 5, where detailed inquiries about their referral practices were made. After this, they moved to Sect. 6 for a deeper dive into data collection, culminating in the form submission stage.

### Data analysis

#### Quantitative data analysis

Data collected through the survey was analyzed using IBM SPSS Statistics version 21. Descriptive statistics, including frequencies and percentages, were computed. The chi-square test was employed to assess the frequency distribution of categorical variables, with a significance level set at less than 0.05 as shown in Tables 3 and 4. Responses were computed in frequencies and percentages as shown in Tables 5, 6, 7, 8, 9, 10 and 11. The data related to the knowledge questions were recoded into categories for analysis. For the convenience of analysis, responses on a five-point Likert scale related to knowledge questions were recoded and re-categorised into three groups. 1—Fully aware merged with aware, 2—Neutral was kept original, 3— Not fully aware was merged with not aware.

**Table 3:** Participants’ responses to knowledge questions (208)

To what extent were you aware that physical therapists can treat patients with TMD by reducing jaw movements and restoring masticatory muscle function?	Not Aware *N*(%)	Neutral *N*(%)	Aware *N*(%)	*P* value
Bachelors	26 (26)	28 (28)	74 (74)	0.219
PG DIP	0 (0)	4 (4)	14 (14)	
Masters	16 (16)	14 (14)	30 (30)	
Doctorate	0 (0)	0 (0)	2 (2)	
Total	42 (20.19)	46(22.11)	120(57.69)	
To what extent were you aware that cervical spine pain may be involved as a cause of masticatory region pain?	Not Aware N(%)	Neutral N(%)	Aware N(%)	*P* value
Bachelors	40 (40)	36 (36)	52 (52)	0.031
PG DIP	0 (0)	4 (4)	14 (14)	
Masters	18 (18)	12 (12)	30 (30)	
Doctorate	0 (0)	0 (0)	2 (2)	
Total	58(27.88)	52(25)	98(47)	
To what extent were you aware that physical therapy can improve TMD symptoms with oral exercises, manual therapy, and postural reduction?	Not Aware N(%)	Neutral N(%)	Aware N(%)	*P* value
Bachelors	16 (16)	20 (20)	92 (92)	0.470
PG DIP	2 (2)	2 (2)	14 (14)	
Masters	14 (14)	10 (10)	36 (36)	
Doctorate	0 (0)	0 (0)	2 (2)	
Total	32 (15.38)	32 (15.38)	144(69.23)	

**Table 4:** Participants responses to questions related to TMD patient population (198)

Do any of your patients with TMD present with neck pain during evaluation?				
	Yes N(%)	No N(%)	Never Evaluated N(%)	*P* value
Bachelors	57 (57)	42 (42)	22 (22)	0.235
PG DIP	12 (12)	5 (5)	0 (0)	
Masters	29 (29)	16 (16)	13 (13)	
Doctorate	2 (2)	0 (0)	0 (0)	
Total	100 (50)	63 (31)	35 (17)	
Does any of your patients with TMD present with cervicogenic headache during evaluation?				
	Yes N(%)	No N(%)	Never Evaluated N(%)	*P* value
Bachelors	48 (48)	54 (54)	19 (19)	0.534
PG DIP	8 (8)	5 (5)	4 (4)	
Masters	25 (25)	26 (26)	7 (7)	
Doctorate	2 (2)	0 (0)	0 (0)	
Total	83 (41)	85(42)	30 (15)	
Do any of your patients with TMD present with poor posture during evaluation?				
	Yes N(%)	No N(%)	Never Evaluated N(%)	*P* value
Bachelors	46 (46)	47 (47)	28 (28)	0.009
PG DIP	10 (10)	7 (7)	0 (0)	
Masters	37 (37)	14 (14)	7 (7)	
Doctorate	2 (2)	0 (0)	0 (0)	
Total	95 (47)	68 (34)	35 (17)	

**Table 5:** Demographic overview of the participants categorized by gender and educational attainment

Gender	Bachelors *N*(%)	PG DIP *N*(%)	Masters *N*(%)	Doctorate *N*(%)	Total *N*(%)
Male	50 (22.5)	10(4.5)	46 (20.7)	2 (0.9)	108 (48.6)
Female	77 (39.6)	8 (3.6)	16 (7.2)	2 (0.9)	114 (51.4)
Total	138 (62.2)	18 (8.1)	62 (27.9)	4(1.8)	222 (100)

**Table 6:** Types of TMD evaluated and treated by practitioners in Karachi N(198)

	TMJ Disc Displacement *N* (%)	TMJ Degeneration *N* (%)	TMJ Hypermobility *N* (%)	TMJ Hypomobility *N* (%)	Myofascial pain *N* (%)	Parafunctional Habits *N* (%)
Bachelors	43 (53.8)	16 (50)	14 (35)	76 (65)	72 (59)	79 (61.2)
PG DIP	10 (12.5)	6 (18.8)	10 (25)	12 (10.3)	9 (7.4)	11 (8.5)
Masters	25 (31.2)	8 (25)	14 (35)	27 (23.1)	39 (32)	37 (28.7)
Doctorate	2 (2.5)	2 (6.2)	2 (5)	2 (1.7)	2 (1.6)	2 (1.6)
Total	80(100)	32(100)	40(100)	117(100)	122(100)	129(100)

Participants can choose more than one option.

**Table 7:** Methods used by practitioners to treat patients with temporomandibular disorder (TMD) N(198)

	Bite splint/ Occlusal guards *N* (%)	Occlusion correction *N* (%)	Medication *N* (%)	Cause-specific treatment *N* (%)
Bachelors	59 (56.7)	36 (54.5)	73(65.2)	77 (65.3)
PG DIP	7 (6.7)	4 (6.1)	7 (6.3)	11 (9.3)
Masters	36 (34.6)	24 (36.4)	30 (26.8)	30 (25.4)
Doctorate	2 (1.9)	2 (3.0)	2 (1.8)	0 (0)
Total	104(100)	66(100)	112(100)	118(100)

Participants can choose more than one option.

**Table 8:** Distribution of participants referring patients to physiotherapist(*N* = 62)

Gender	*N* (%)
Female	27 (43.5)
Male	35 (56.4)
Highest Level of Degree	
Bachelor’s degree	33 (53.2)
Master’s degree	21 (33.8)
Post Graduate Diploma	6 (9.6)
Doctorate	2 (3.2)

**Table 9:** Practitioners reasons for not referring patients with TMD to physiotherapist (*N* = 136)

	Lack of collaboration *N* (%)	Lack of awareness *N* (%)	Limited access *N* (%)	No Need for a Physical Therapist *N* (%)	Other *N* (%)
Bachelors	77 (61.6)	70 (58.3)	36 (54.5)	73(65.2)	0 (0)
PG DIP	11 (8.8)	5 (4.2)	4 (6.1)	7 (6.3)	2 (50)
Masters	35 (28)	43 (35.8)	24 (36.4)	30 (26.8)	2 (50)
Doctorate	2 (1.6)	2 (1.7)	2 (3.0)	2 (1.8)	0 (0)
Total	125(100)	120(100)	66(100)	112(100)	4(100)

Participants can choose more than one option.

**Table 10:** Reasons practitioners referring patients with temporomandibular disorder (TMD) to a physiotherapist (*N* = 62)

	Neck pain *N* (%)	Postural alteration *N* (%)	Masticatory muscle tenderness *N* (%)	Headache *N* (%)
Bachelors	31 (64.5)	24 (68.6)	26 (68.4)	21 (67.7)
PG DIP	6 (12.5)	4 (11.4)	4 (10.5)	4 (12.9)
Masters	9 (18.7)	7 (20)	8 (21.1)	6 (19.4)
Doctorate	2(4.1)	0(0)	0(0)	0(0)
Total	48 (100)	35 (100)	38 (100)	31 (100)

Participants can choose more than one option.

**Table 11:** Need for more learning about the benefits of collaboration with physical therapists to treat patients with TMD (*N* = 198)

	Disagree *N* (%)	Not sure *N* (%)	Agree *N* (%)
Bachelors	1 (0.5)	3 (1.5)	117(59.1)
PG DIP	0	3 (1.5)	14 (7.1)
Masters	1 (0.5)	1 (0.5)	56 (28.3)
Doctorate	2 (1)	1 (0.5)	1 (0.5)
Total	2 (1.0)	8 (4.0)	188 (94.9)

#### Qualitative data analysis

**Fig. 2:**
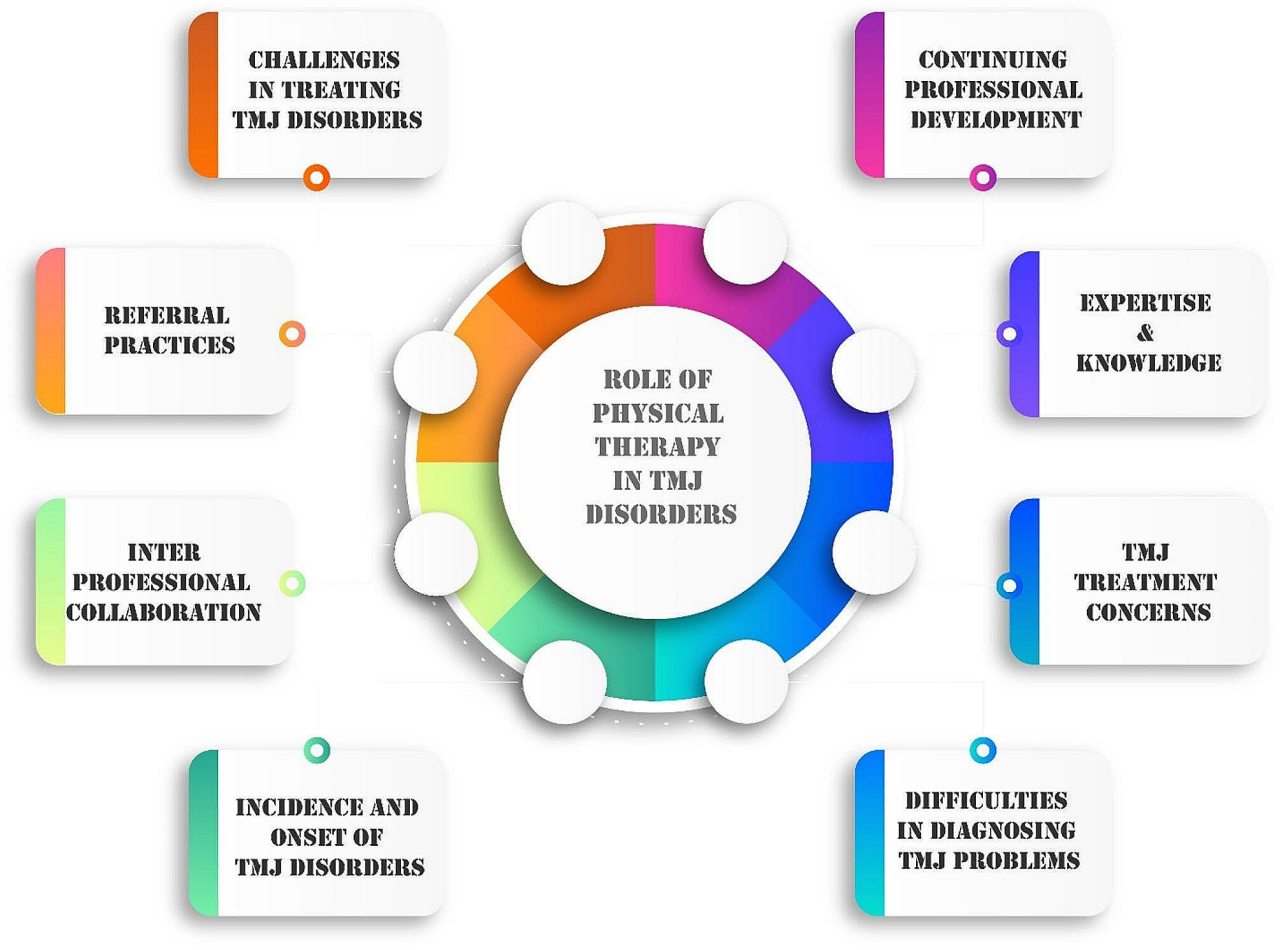
Eight Themes related to the role of physical therapy in TMJ disorders

Qualitative data were analyzed using thematic analysis. Common themes, patterns, and insights were identified in the interview responses, aligning with the study objectives. The analysis of the interviews was performed simultaneously with the inductive interpretation of the data. All interviews were recorded using Audacity software, which was used to eliminate pauses and enhance audio quality. These recordings were then converted to mp3 format and transcribed. A manual analysis of these transcriptions followed, from which themes and codes were derived, as depicted in Tables 11 and 12. The key sentences representing each category were then selected from the transcripts. The data interpretation and coding were collaboratively conducted by all authors, with themes being finalized through collective discussion. Throughout the coding process, data accumulation and thematic analysis were conducted. This included a constant comparative analysis, adhering to grounded theory principles, where themes were continually compared against the data. During this phase, disconfirmatory evidence was actively sought in subsequent interviews, particularly after establishing thematic categories in the initial ones. The data from each group substantiated a set of master themes, as indicated in Tables 12 and 13; Fig. 2.

The decision on the number of interviews was based on achieving thematic saturation, a point at which new data no longer contribute significantly to the findings due to repetitive themes and responses of the participants. This marked the end of the data generation process. The final sample included 20 dentists. The thematic analysis then focused on pre-existing codes derived from these 20 participants. Both quantitative and qualitative data were then integrated and analyzed comprehensively.

**Table 12:** Outlines the themes, sub-themes, codes, and representative sentences

Theme	Sub-theme	Code	Code Number	Representative Sentence(s)
Incidence and Onset of TMJ Disorders in Dental Practice	Frequency	Rare Encounters	01	“TMJ problems aren’t encountered very frequently in our clinic.”
	Context of Occurrence	Procedure-Induced Symptoms	02	“Others may develop symptoms during dental procedures…”
Difficulties in diagnosing TMJ problems	Diagnostic Practice	Palpation	03	“I typically start by palpating the area in front of the ear…”
	Diagnostic Indicators	Symptoms	04	“If there’s limited mouth opening, trismus, or pain during the jaw’s movement…”
Challenges in treating TMJ disorders	First Line of Treatment	Analgesics	05	“I usually prescribe analgesics, such as NSAIDs, or apply topical NSAIDs for relief.”
	Clinical Efficacy and Practitioner Challenges	Perceived Treatment Ineffectiveness and Practitioner Frustration	07	“The clicking sound never goes no matter what I do !” “I seem helpless in treating TMD problems in certain patients as nothing works!”
	Complexity in Management and Treatment of TMD.	Inadequacy of Conventional Therapeutics and Shift to Pharmacological Adjunct	15,17	“TMJ problems are chronic and difficult to manage.” “….TMD problems are notoriously difficult to treat, medications and analgesic gels don’t help much, and we end up giving benzodiazepines and sleep medications.”
Expertise and Knowledge	Self-assessment	Limited Knowledge	08	“I have very little knowledge and no expertise in this area of TMJ problems.”
	Reason for Limitation	Insufficient Exposure	09	“This is due to insufficient exposure during both my undergraduate and postgraduate training.”
Referral Practices	Referral for Non-TMJ Issues	Physiotherapy	10	“Yes, I have referred patients to physiotherapists for neck pain or related problems.” “I consider patient needs and the severity of TMD symptoms when making referrals.”
	Referral for Special Cases	Specialist Referral	11	“When it comes to TMJ pain dysfunction syndrome, I usually refer the patient to a specialist.” “I refer them to an oral surgeon that I trust.”
Continuing Professional Development (CPD)	CPD on TMJ Problems	Insufficient CPD	12	“No, there aren’t many CPD programs that address TMJ issues adequately…”
	Curriculum and Exposure	Lack of knowledge of the role of physiotherapists in TMJ treatment	13	“It is not sufficiently covered in the undergraduate curriculum, and there is not enough exposure afterward either.”
Interprofessional Collaboration	Collaboration with Physiotherapists	None	14	“No, I have no collaboration with physiotherapists.”
TMJ Treatment Concerns	Dental Treatment Priority	Avoiding Interference	16	“My primary concern is ensuring that these problems do not interfere with the dental treatment I provide.”

**Table 13:** Code numbers and descriptions

Number	Code Description
01	Rare encounters with TMJ problems in the dental clinic.
02	Occurrence of TMJ symptoms during dental procedures.
03	Use of palpation in front of the ear to diagnose TMJ problems.
04	Identification of TMJ problems by symptoms such as limited mouth opening, trismus, or pain.
05	Prescription of analgesics such as NSAIDs for TMJ treatment.
06	Referral of patients to oral surgeons for TMJ issues.
07	Non-utilization of specific TMJ treatments like occlusal splints or night guards.
08	Dentist’s self-assessment of having limited knowledge and expertise in TMJ problems.
09	Lack of exposure to TMJ problems during undergraduate and postgraduate education.
10	Referral of patients to physiotherapists for conditions other than TMJ issues.
11	Referral of patients to specialists for conditions like TMJ pain dysfunction syndrome.
12	Perception of insufficient continuing professional development programs addressing TMJ problems.
13	Inadequate coverage of TMJ issues in the dental curriculum and postgraduate exposure.
14	Absence of collaboration with physiotherapists in treating TMJ problems.
15	Recognition of TMJ problems as chronic and difficult to manage.
16	Concern for dental treatments to not be interfered with by TMJ problems.
17	Need to shift from conventional TMD treatments to the use of pharmacological alternatives when initial therapies prove ineffective.

## Results

### Questionnaire validation

The reliability of the questionnaire was evaluated using Cronbach’s Alpha. A total of 28 items, spread over three domains, were assessed for reliability; the items were reliable (Table 1). The set of items that evaluated knowledge and referral practices yielded a Cronbach alpha value of 0.65 and 0.67, suggesting moderate reliability. On the other hand, the population of patients with items assessing the temporomandibular disorder (TMD) demonstrated a Cronbach’s alpha value of 0.81, suggesting a high level of reliability.

Table 2 presents Pearson correlation coefficients for questions concerning temporomandibular disorders (TMD) and physical therapy awareness. Correlations were evaluated against a critical value of 0.32 when analysing the data with two degrees of freedom.

#### Awareness about physical therapists in treating TMD

The answers to the first three questions are based on the prior knowledge of the specific aspects of physical therapy treatment of TMD. A high correlation coefficient (0.815, 0.742, 0.771) and highly significant *p*-values (0.001) were obtained for all three questions.

#### Types of TMD evaluated and treated

The respondents were asked what types of TMD they had evaluated and treated. These include TMJ disc displacement, TMJ degeneration, TMJ hypermobility, TMJ hypomobility, myofascial pain, and parafunctional habits. Various levels of significance were indicated by *p*-values for each of these correlation coefficients, ranging from 0.330 to 0.587.

#### Evaluation methods for TMD patients

An inquiry was made regarding the methods used for evaluating TMD patients in the survey. There were robust correlations between these methods (ranging from 0.484 to 0.747), all with significant *p*-values (0.001 to 0.002).

#### Co-occurrence of TMD with other symptoms

Questions regarding the presence of neck pain, headache, and poor posture during TMD patients’ evaluation were also included. Statistical significance was indicated by moderate to high correlation coefficients (0.334 to 0.635), with *p*-values ranging from 0.001 to 0.041.

#### Treatment methods for TMD

Participants were asked to describe their TMD treatment methods, including bite splints, occlusal guards, occlusion correction/braces, prescription medications, and cause-specific treatments. Significant *p*-values were associated with the correlation coefficients for each of these methods, ranging from 0.473 to 0.588.

#### Reasons for referral to a physical therapist

Final questions focused on reasons to refer a TMD patient to a physical therapist, such as neck pain, postural changes, masticatory muscle tenderness, and headaches. These questions revealed strong correlations (0.487 to 0.737) and highly significant *p*-values (0.001 to 0.002).

The questions across all domains of the questionnaire exhibit significant positive correlations, indicating that these questions were effective.

A total of 222 participants filled out the questionnaire out of 332, with a response rate of 66%. Table 5 showcases the distribution of participants based on gender and their respective educational achievements. From the total pool of 222 respondent’s slight majority were female, comprising 51.4% (114 participants), while males constituted 48.6% (108 participants).

Regarding educational attainment, a bachelor’s was the predominant qualification, with 62.2% of the participants holding this degree. Interestingly, a significant majority of those with a bachelor’s degree were females, representing 39.6% of the total participants, compared to 22.5% being males.

Masters was the next most common qualification, held by 27.9% of the respondents. Here, the gender distribution was skewed towards males, with 20.7% of the total cohort being male master holders, as opposed to 7.2% females. PG DIP was held by 8.1% of the participants. The gender split was relatively even, with males at 4.5% and females at 3.6% of the total sample. The least common qualification was the Doctorate, with only 1.8% of the participants holding this degree. The gender distribution was evenly split, with males and females each constituting 0.9% of the total participants.

### Dentist knowledge of physical therapy in TMD Treatment

Table 3 shows practitioners’ knowledge about various aspects of Temporomandibular Disorders (TMD) and the role of physical therapy in its management. The majority, 57.69% (120 participants), were aware that physical therapists can treat patients with TMD by reducing jaw movements and restoring masticatory muscle function. 22.11% (46 participants) remained neutral on this matter. Only 20.19% (42 participants) were not aware of this capability. The difference in knowledge across educational levels for this aspect was not statistically significant, with a *p*-value of 0.219.

Nearly half, 47% (98 participants), were aware that cervical spine pain might be involved as a cause of masticatory region pain. The difference in knowledge between educational levels for this aspect was statistically significant, with a *p*-value of 0.031, indicating that one’s educational attainment could influence one’s awareness of this subject.

A significant majority, 69.23% (144 participants), were cognizant that physical therapy can enhance TMD symptoms through oral exercises, manual therapy, and postural reduction. The difference in knowledge across educational levels for this topic was not statistically significant, with a *p*-value of 0.470.

Table 4 elucidates the responses of practitioners regarding the presentation of certain symptoms in their patients with Temporomandibular Disorders (TMDs). Half of the practitioners, 50% (100 out of 198), reported that their TMD patients exhibited neck pain during the evaluation. The variation in responses based on educational qualifications was not statistically significant, as indicated by a *p*-value of 0.235.

A total of 41% (83 out of 198) of the practitioners observed their TMD patients presenting with cervicogenic headaches during the evaluation. A slightly higher proportion, 42% (85 out of 198), did not note this symptom, while 15% (30 out of 198) had never evaluated the presence of cervicogenic headaches in their patients. The *p*-value of 0.534 suggests that the observed variations across educational backgrounds were not statistically significant.

A total of 47% (95 out of 198) of the practitioners indicated that poor posture was evident in their TMD patients during the evaluation. 34% (68 out of 198) did not observe any postural issues in their patients. The remaining 17% (35 out of 198) had never assessed this symptom. The differences in observations, especially related to posture, were found to be statistically significant across the educational qualifications, as indicated by a *p*-value of 0.009.

The data highlights the diverse symptomatology exhibited by TMD patients. Although neck pain and poor posture were relatively common findings, the presentation of cervicogenic headaches was almost equally observed and unobserved among practitioners. Educational background, particularly in the context of postural evaluation, appeared to influence the observations of the practitioners.

### Types of TMDs treated in dental clinics

Table 6 delineates the types of Temporomandibular Disorders (TMD) practitioners in Karachi have evaluated and treated, segmented by their educational qualifications. It is essential to note that participants had the flexibility to select multiple options, indicating their experience in evaluating and treating various TMD types. Among the types of TMD, TMJ hypomobility and myofascial pain were the most evaluated and treated by the practitioners. Most practitioners holding a bachelor’s degree treated TMJ hypomobility (65%), myofascial pain (59%), and parafunctional Habits (61.2%). Meanwhile, those with a master’s qualification also showed a significant interest in myofascial pain (32%) and parafunctional Habits (28.7%). Practitioners with PG DIP and Doctorate qualifications were less represented in all TMD categories.

Table 7 displays data on the treatment methods practitioners employ for Temporomandibular Disorder (TMD) patients in Karachi. It’s important to note that participants could select multiple treatment methods, meaning a single practitioner might employ several methods to treat TMDs. In summary, most practitioners holding a bachelor’s degree predominantly used medication (65.2%) and cause-specific treatment (65.3%) as their treatment methods for TMD. Bite splint/occlusal guards and occlusion correction were also commonly used by this group, with 56.7% and 54.5%, respectively. Practitioners with a master’s qualification also had a significant representation in all treatment methods, notably using Bite splint/occlusal guards (34.6%) and occlusion correction (36.4%). Practitioners with PG DIP and Doctorate qualifications had a lesser representation across all treatment methods, with those holding a Doctorate not employing cause-specific treatment.

### Dentist-physiotherapist collaboration in TMD Management: an examination of referral practices

In exploring dentists’ practices concerning the referral of temporomandibular joint disorder (TMD) patients to physiotherapists, our survey, as encapsulated in Table 8, illuminates intriguing patterns among the 62 participating dental professionals.

Gender-wise, the landscape of referrals mirrors a nuanced balance, albeit with a slight tilt. Among our respondents, male dentists hold a slender majority. They constituted 35 participants, translating to 56.4% of the sample. Female dentists, contributing a significant 43.5%, are represented by 27 respondents. This gender distribution not only reflects the evolving dynamics in dental practice but also underscores the inclusive nature of the profession when it comes to managing TMD.

Exploring their academic qualifications reveals a significant diversity in educational backgrounds among these dentists. The majority, over half of the respondents (53.2%), have completed a bachelor’s degree. This suggests that foundational dental education significantly emphasizes the multidisciplinary approach to TMD, including physiotherapy referrals. Following closely, the holders of master’s degrees make up 33.8% of our sample, indicating that advanced studies in dentistry also resonate with the importance of physiotherapy in TMD management.

Interestingly, the survey also captures a segment of the dental fraternity with even higher qualifications. Post Graduate Diploma holders, who account for 9.6% of the respondents, and the 3.2% who have attained a Doctorate, collectively reinforce the notion that across varied levels of academic achievement, there is a consistent recognition of the role of physiotherapy in treating TMD.

Table 9 presents the reasons given by practitioners for not referring patients with temporomandibular disorders (TMD) to a physiotherapist, segmented by their educational qualifications. Bachelor’s degree holders consistently formed the majority for most reasons, indicating they might be the most populous group among the participants. “Lack of Collaboration” and “No Need for Physical Therapist” were the predominant reasons across all education levels, especially those with bachelor’s degrees. The most commonly cited reasons were a lack of collaboration and a perceived lack of need for a physical therapist, especially among those with a bachelor’s degree.

Table 10 showcases data on the reasons practitioners refer patients with temporomandibular disorder (TMD) to a physiotherapist. It is emphasized that participants could select more than one reason, meaning a single practitioner might refer for multiple reasons. Most referrals to physiotherapists for patients with TMD were made by practitioners holding a bachelor’s degree. These practitioners most frequently referred for neck pain (67.4%), postural alteration (68.6%), masticatory muscle tenderness (68.4%), and headache (67.7%). Practitioners with a master’s degree also had a notable representation in all referral reasons, with percentages ranging from 19.4 to 21.1%. Those with a PG DIP qualification had a consistent representation across all reasons for referral, with percentages hovering around 10–13%.

Table 11 presents data on practitioners’ perceived need for further learning regarding the advantages of collaborating with physical therapists in treating patients with temporomandibular disorder (TMD). The data shows that an overwhelming majority (94.9%) of the practitioners across all educational levels believe there is a need for more learning about the benefits of collaboration with physical therapists in treating TMD patients. Specifically, the highest agreement was observed among those with a bachelor’s degree (59.1%). Practitioners with a master’s degree also significantly agreed, constituting 28.3% of the total.

Table 12 outlines the themes, sub-themes, codes, and representative sentences extracted from the interview transcript using thematic analysis as per the article by Terry et al. [25]. Each code was given a number for easy reference, and the sentences represent the respective codes.

In the qualitative analysis of the interview transcript regarding temporomandibular joint (TMJ) problems encountered in dental practice, several themes and codes that align with current literature on the subject have emerged, as shown in Fig. 2.

### Incidence and onset of TMJ disorders

“TMJ problems aren’t encountered very frequently in our clinic.” (01GD).

The infrequent encounter of TMJ problems in general dental practice is supported by the literature, which suggests that while TMJ disorders are common, not all cases require intervention by a dentist [26].

“Others may develop symptoms during dental procedures…” (02PGD).

The sub-theme of Procedure-Induced Symptoms underscores the notion that dental procedures themselves can precipitate or exacerbate TMJ symptoms [27]. This highlights a gap in understanding patient-specific risk factors for TMJ disorders that could be exacerbated by dental treatments.

### Difficulties in diagnosing TMJ problems

The diagnostic practices such as palpation are consistent with the recommendations of the American Dental Association (ADA), though they raise concerns about the subjectivity and variability of diagnostic techniques [28]. Symptoms such as pain and trismus are well-documented indicators of TMJ disorders [29], but they point to the need for more objective and standardized diagnostic criteria.

The diagnostic approach described by the dentist, involving palpation, and identifying symptomatic indicators, is consistent with clinical guidelines that recommend a non-invasive approach as the first line of TMJ disorder diagnosis [30].

### Challenges in treating TMJ disorders

#### First line of treatment

The use of NSAIDs for the treatment of TMJ problems (Code 05) is widely supported as an initial management strategy for pain relief [31]. The reluctance to use more involved treatment modalities, such as occlusal splints (Code 07), may reflect a conservative treatment philosophy, which is encouraged by research that has found inconsistent results with such devices [32].

#### Clinical efficacy and practitioner challenges

The thematic analysis of clinical efficacy and practitioner challenges in managing temporomandibular joint disorders (TMJ/TMD) uncovers significant issues faced by dental professionals. These challenges are rooted in the perceived ineffectiveness of treatments and the complex nature of TMD management.

The subtheme of “Perceived Treatment Ineffectiveness and Practitioner Frustration” (Code 07) reflects a widespread sentiment among dental practitioners. The frustration expressed over the persistence of symptoms like the clicking sound, despite treatment efforts, aligns with findings in the literature. For example, Afroz et al. (2018), in a systematic review, noted that the multifactorial etiology of TMD makes it challenging to achieve consistent treatment outcomes [33]. This complexity often results in a trial-and-error approach, which can be disheartening for both practitioners and patients.

Furthermore, practitioners feeling ‘helpless’ in treating TMD aligns with research indicating that the variability in TMD symptoms and responses to treatment can lead to practitioner uncertainty [34]. This situation is compounded by the lack of a universally accepted treatment protocol for TMD, as noted by Schiffman et al. (2014), making the management of these disorders particularly challenging [8].

#### Complexity in management and treatment of TMD

The “Inadequacy of Conventional Therapeutics and Shift to Pharmacological Adjunct” (Codes 15 and 17) subtheme highlights another critical aspect of TMD management. The ineffectiveness of standard medications and analgesic gels in providing lasting relief is a known issue, as identified by Manfredini and Stellini (2009) [27]. Their findings suggest that while initial conservative treatments like NSAIDs and physical therapy are recommended, they often do not suffice for all patients, leading to the exploration of alternative treatments.

The reliance on benzodiazepines and sleep medications, as mentioned in the representative sentences, underscores the complexity of TMD, often interlinked with factors such as stress and poor sleep quality [35]. While these pharmacological adjuncts can provide symptom relief, particularly in cases where stress exacerbates TMD, they are not without risks and do not address the root cause of the disorder [36].

The shift to these adjunctive therapies could be indicative of a broader trend in TMD management, where a multidisciplinary approach is increasingly recognized as necessary [21]. This approach may involve collaboration with mental health professionals, sleep specialists, and physiotherapists, highlighting the need for comprehensive patient care beyond the scope of traditional dentistry.

#### Expertise and knowledge

The dentist’s admission of limited knowledge and expertise in treating TMJ problems (Code 08) could reflect a broader issue within dental education, where insufficient emphasis is placed on TMJ disorders [37]. This corresponds with the reported lack of exposure during training (Code 09), suggesting a need for enhanced educational modules on TMJ disorders within dental curricula [38].

#### Referral practices

The preference for referral to oral surgeons (Code 06) is a common practice, especially in complex cases or when the general dentist’s expertise in TMJ disorders is limited [30]. However, the lack of referral to physiotherapists (Code 10) could be seen as a missed opportunity, as physiotherapy has been shown to be effective in the management of TMJ disorders, particularly when combined with dental treatments [39].

#### CPD on TMJ problems

The thematic analysis focusing on Continuing Professional Development (CPD) and Curriculum and Exposure regarding temporomandibular joint (TMJ) problems highlights significant gaps in current dental education and ongoing professional training. These gaps have substantial implications for the quality of care provided to patients with TMJ disorders.

The sub-theme of “Insufficient CPD” (Code 12) aligns with the broader concerns in the dental community about the adequacy of ongoing education related to TMJ disorders. The sentiment that “there aren’t many CPD programs that address TMJ issues adequately” is corroborated by recent literature [40]. It has been noted in several studies that despite the prevalence of TMJ disorders, many dentists feel underprepared to manage these conditions due to insufficient training [41]. This lack of preparedness can lead to suboptimal patient care and a reliance on outdated or ineffective treatment modalities.

The inadequacy of CPD programs is a critical issue, considering the evolving nature of TMJ disorder management. As novel treatment approaches and research findings emerge regularly, CPD programs are vital for dental practitioners to stay abreast of these developments [42]. The current gap in CPD offerings not only hinders dentists’ ability to provide evidence-based care but also impacts their confidence in managing these complex cases [43].

#### Curriculum and exposure

Regarding the “Lack of knowledge of the role of physiotherapists in TMJ treatment” (Code 13), the concern raised about insufficient coverage in the undergraduate curriculum and lack of exposure afterward points to a systemic issue in dental education. This gap is particularly significant given the interdisciplinary nature of TMJ disorder management. The role of physiotherapists in treating TMJ disorders, as highlighted in studies like McNeely et al. (2006), indicates that an understanding of physiotherapy’s role is crucial for comprehensive patient care [13]. However, if this aspect is not adequately covered in educational programs, dentists may be less likely to refer patients to physiotherapy or collaborate effectively with these professionals.

The lack of emphasis on interdisciplinary approaches in dental education is a missed opportunity, as collaborative care is increasingly recognized as beneficial for TMJ disorder management. Research by List and Axelsson (2010) highlights the importance of an integrated approach, suggesting that patients often require a combination of dental and physiotherapy interventions for optimal outcomes [22]. Therefore, current curriculum limitations, especially in the context of Pakistan, may inadvertently perpetuate a siloed approach to care, which is less effective in managing multifactorial conditions such as TMJ disorders.

#### TMJ treatment concerns

The theme “TMJ Treatment Concerns,” specifically under the subtheme of “Dental Treatment Priority” with a focus on “Avoiding Interference” (Code 16), reveals a significant aspect of clinical practice where dentists prioritize the smooth execution of dental procedures, ensuring that temporomandibular joint (TMJ) disorders do not disrupt their primary treatment goals. The representative sentence, “My primary concern is ensuring that these problems do not interfere with the dental treatment I provide,” reflects a pragmatic approach in clinical settings, which aligns with and raises questions about the existing literature on TMJ disorder management [8].

#### TMJ treatment concerns and dental practice

The pragmatic approach of prioritizing dental procedures while managing TMJ disorders is a common thread in dental practice. Numerous studies have highlighted that dentists often focus on the primary dental issues at hand, treating TMJ symptoms as secondary or referring them out when they pose a risk to dental treatment [8, 14]. This practice may be partly due to the complexities involved in TMJ disorder management and the need for specific expertise [22].

#### Balancing TMJ management and dental procedures

The challenge for dentists lies in balancing the management of TMJ disorders with other dental treatments. Studies have indicated that while TMJ disorders can significantly impact dental treatment outcomes, there is a tendency in general dental practice to compartmentalize these issues [30]. This separation may lead to a scenario where TMJ disorders are not addressed in oral health, potentially overlooking the interconnectivity of oral structures and functions.

#### Concerns about interference with dental treatments

The concern about TMJ disorders interfering with dental treatments is not unfounded. As Schiffman et al. (2014) indicated, TMJ disorders can lead to complications such as limited mouth opening or discomfort during procedures, which can directly affect the delivery of dental care [8]. This situation necessitates a careful assessment and management strategy to ensure that both TMJ disorders and other dental needs are addressed effectively.

#### Need for integrated management

Given these challenges, there is growing advocacy for more integrated management approaches. For example, the work of List and Axelsson (2010) suggests incorporating the assessment of TMJ disorder as a routine part of dental examinations. Such integration ensures that TMJ issues are identified early and managed in conjunction with other dental treatments rather than being treated as separate or secondary concerns [22].

#### Integration of quantitative and qualitative findings

The findings from the quantitative and qualitative components of the study were integrated to provide a comprehensive understanding of dentists’ knowledge, referral practices, and experiences related to TMD and physical therapy.

By adopting this mixed-method approach, the study aimed to provide a holistic perspective on the study objectives, combining quantitative data with rich qualitative insights from in-depth interviews with dentists.

The combined qualitative and quantitative findings of the study on temporomandibular disorders (TMD) among practitioners offer a comprehensive view of the current state of knowledge, management practices, and attitudes toward interdisciplinary collaboration in TMD treatment.

From a qualitative standpoint, themes such as the infrequent encounter of TMJ problems in general dental practice, challenges in diagnosing and treating TMJ disorders, and the need for enhanced educational modules on TMJ disorders within dental curricula were prominent. Dentists expressed concerns about the subjectivity in diagnosis and a preference for conservative treatment approaches. They also highlighted the complexity of TMD management and the importance of incorporating multidisciplinary strategies, including physiotherapy.

According to the present study, there was no statistically significant difference among educational levels regarding knowledge of physical therapy’s role in TMD treatment. In contrast to their higher-qualified counterparts, bachelor’s degree holders referred more patients to physiotherapists. This finding suggests that non-surgical, interdisciplinary treatment approaches for TMD are becoming more prevalent.

Most practitioners also acknowledged the need for further education about the benefits of physiotherapy in treating TMDs. Despite this recognition, barriers to interdisciplinary collaboration were observed, such as a lack of collaboration and the perceived absence of physical therapy.

The most common treatments used by the respondents were medications and bite splints, consistent with previous studies. In contrast, a substantial number of practitioners incorporated physiotherapy interventions, indicating a trend toward more conservative and functional approaches to treatment.

Overall, the study highlights the evolving landscape of TMD management in dental practice. It highlights the need for improved education and training in TMD, greater awareness and utilization of physiotherapy, and the importance of interdisciplinary collaboration to enhance patient care.

## Discussion

Recent data highlighted that most dentists are unaware of the benefits of collaborating with physiotherapists [13, 21, 22]. To improve patient care, this knowledge gap must be addressed. The lack of comprehensive data on dentists’ awareness, especially in Karachi, indicates the urgency of addressing this awareness gap. To address this gap, the present study aims to (1) validate a knowledge assessment tool. (2) Assessing dentists’ knowledge about physical therapy’s importance in treating TMD patients using a knowledge assessment tool. (3) To determine the extent to which dentists collaborate with physiotherapists for TMD management through referral practices. This aspect of collaboration was measured by collecting data on dentist-physiotherapist collaboration. (4) To investigate the types of TMD treated in Karachi dental clinics. (5) Conduct interviews with dentists to gain insight into treatment, knowledge, and referral practices related to TMD and physical therapy as a treatment option.

The primary objective of this study was to validate an adopted questionnaire for use in Pakistan, a region where no validated questionnaire had been reported prior to the study. Our findings confirm that the current questionnaire is both reliable and valid, making it a valuable tool for assessing dentist knowledge regarding the importance of physical therapy in treating patients with TMDs in Pakistani populations.

The questionnaire demonstrated good internal consistency, as indicated by Cronbach’s alpha of 0.67, 0.81 and 0.65 in three domains of the questionnaire. In terms of construct validity, the strong positive correlations between questions in all domains of the questionnaire indicate that the items effectively capturing the essence as intended. The correlation values exceeding the critical value of 0.32 further emphasize the meaningfulness of these correlations. These findings are crucial because they underscore the ability to distinguish between individuals with varying degrees of knowledge based on their responses. However, the results cannot be compared because no previous study has provided information about the validity of the questionnaire [24].

Our study revealed a nuanced understanding of the role of gender and educational attainment in the knowledge and management of temporomandibular disorders (TMD) among practitioners. Notably, a slight majority of females among our respondents reflect similar gender distributions in related research, underscoring the active engagement of women in the healthcare sector [44].

Our findings align with Farooq et al. [23], who reported a spectrum of awareness levels among dental practitioners regarding the role of physiotherapy in managing TMD. While our study found no significant knowledge difference across educational levels concerning the role of physical therapy in TMD treatment (*p* = 0.219), Farooq et al. highlighted a general lack of awareness that could be attributed to suboptimal referral rates to physiotherapy services [23].

The knowledge about the role of physiotherapy in TMD management among practitioners in the survey appears to be more widespread compared to Ahmed’s study [5, 45], where a significant portion of dentists lacked such knowledge before the survey was conducted. However, both studies reveal an eagerness among dentists to learn more about the benefits of physiotherapy, with the survey indicating that nearly all participants recognized the need for further learning. In terms of educational qualifications, the survey identified a higher prevalence of bachelor’s degree holders, consistent with Ahmed’s findings, where maxillofacial surgeons and master’s degree holders demonstrated greater awareness of physiotherapy management for TMD [45]. This highlights a potential knowledge gap among practitioners with different levels of education.

The referral patterns to physiotherapists in our study are consistent with those in the literature, where a predilection for referrals to maxillofacial surgeons over physiotherapists was evident [45]. This preference could stem from traditional perspectives on TMD management, which historically prioritized surgical interventions. Our study adds to this discourse by illustrating how educational attainment could influence referral patterns, with those who hold bachelor’s degrees being more likely to refer to physiotherapists compared to their counterparts with higher qualifications.

In the present study, a majority were aware of the potential for PT to treat TMD, which is a more positive finding than in the study conducted by dentists in Saudia [46], where initially, only a small percentage referred patients to PT. Similarly, a study on dental practitioners in Karachi [45] reported a significant reluctance to refer patients. In contrast, our survey suggests a more balanced view, with a majority acknowledging the role of physiotherapy in reducing jaw movements and enhancing masticatory muscle function.

The reasons for not referring patients to physiotherapists, as noted in the survey, such as lack of collaboration and the perceived absence of a need for a physical therapist, also reflect the concerns raised in the published study. It highlights an ongoing challenge in the interdisciplinary collaboration between dentistry and physiotherapy [47].

Consistent with prior studies [22, 48], our respondents predominantly utilized medication and cause-specific treatments. This treatment preference reinforces the findings by Farooq et al., where medication and bite splints were the primary management tools for TMD [23]. Nevertheless, the substantial percentage of practitioners employing physiotherapy interventions in our study indicates a shift towards more conservative and functional treatment modalities.

A significant aspect of our study was the emphasis on interdisciplinary collaboration. The high percentage of practitioners acknowledging the need for further learning about the benefits of such collaboration (94.9%) echoes the sentiments found in other studies [39, 49]. This collective inclination towards interdisciplinary learning and collaboration is indicative of an evolving healthcare landscape where the integration of various treatment modalities is increasingly valued.

In the qualitative analysis of interviews regarding temporomandibular joint (TMJ) disorders in dental practice, several themes emerged, as shown in Fig. 2. TMJ problems are infrequently encountered in general dental practice but can be exacerbated by dental procedures. Diagnostic practices such as palpation are subjective and vary, indicating the need for more standardized criteria.

Treatment often begins with NSAIDs for pain relief, reflecting a conservative approach due to inconsistent results with more involved treatments such as occlusal splints. Dental practitioners face challenges in treatment efficacy and complexity in managing TMJ disorders, often resulting in frustration and a trial-and-error approach. This complexity requires a multidisciplinary approach involving mental health professionals, sleep specialists, and physiotherapists.

Dentists admit limited knowledge and expertise in treating TMJ problems, reflecting gaps in dental education and training. The preference for referral to oral surgeons over physiotherapists may miss opportunities for effective management. Inadequate continuing professional development (CPD) programs and curriculum gaps in understanding the role of physiotherapists contribute to these issues.

The focus on TMJ disorders in dental education in Pakistan is limited, hindering comprehensive patient care. Dentists often prioritize dental treatments over TMJ management, treating TMJ symptoms as secondary. This compartmentalization overlooks the interconnectedness of oral structures. Integrated management strategies, incorporating assessment of TMJ disorder into routine dental examinations, are advocated to address these challenges effectively.

A key strength of our study is the detailed demographic breakdown, which allows for a nuanced analysis of the impact of gender and educational attainment on TMD knowledge and management practices. In addition to that, we applied a robust methodological approach and comprehensive coverage of the subject matter. Moreover, the questionnaire was validated through a pilot test, ensuring both content relevance and construct validity, supported by a satisfactory Cronbach’s Alpha, indicating moderate to high reliability. Furthermore, the branching logic applied in the survey design enhanced the relevance and integrity of the data.

The study’s insights into the management of temporomandibular joint (TMJ) disorders within a dental clinic setting present several notable limitations that warrant careful consideration. The use of convenience sampling, although practical, may introduce selection bias, limiting the generalizability of the findings to all dentists in Karachi. The reliance on self-reported data could also be subjected to response bias, where participants might provide socially desirable answers. Furthermore, the distribution method through WhatsApp and email could have inadvertently excluded practitioners with limited access to these platforms or those less inclined to participate in online surveys, potentially skewing the sample toward a more technologically savvy demographic.

The element of subjectivity is another critical limitation. The reflections and opinions of the interviewed dentist could carry inherent biases, consciously or unconsciously shaping the portrayal of how TMJ disorders are managed. The responses might be influenced by factors such as the desire to present oneself in a favorable light or the imperfect recall of specific clinical cases, introducing recall bias and social desirability bias into the mix.

The dentist’s self-professed lack of expertise and the limited discussion around continuing professional development (CPD) also suggest a disconnect from the evolving body of knowledge regarding TMJ disorder treatments. Without engagement in comprehensive CPD, the dentist’s practices might not reflect the most current standards, which is a limitation when considering the study’s relevance to current clinical practices.

### Recommendations

In addressing the complex challenges of managing temporomandibular joint (TMJ) disorders within dental practice, a comprehensive and multi-dimensional strategy emerges as essential. Central to this approach is the advancement of dental education. It is envisaged that both undergraduate and postgraduate dental curricula should encompass thorough modules on TMJ disorders, thereby arming future dentists with a deep understanding of these conditions. This educational foundation is further strengthened through advanced targeted training and certifications specifically designed around the nuances of TMJ disorder management.

The journey of learning and adaptation does not end with formal education. Continuous professional development (CPD) plays a pivotal role, offering dentists ongoing exposure to the latest advancements in TMJ diagnosis and treatment. The value of these CPD activities is amplified when they foster interdisciplinary collaboration, bringing together diverse healthcare professionals to enrich patient care [50].

When it comes to treating TMJ disorders, a conservative approach is favored initially. This involves prioritizing pain management and patient education over more invasive procedures. Treatment protocols must not be static, but evolve in response to the latest research, maintaining their effectiveness while remaining minimally invasive [51].

A key component of effective TMJ management is the establishment of efficient referral networks. These networks should extend their reach beyond traditional confines, involving not just oral surgeons, but also physiotherapists and other specialists proficient in TMJ disorders. Such collaborative networks are instrumental in providing patients with comprehensive and holistic care, especially with complex TMJ issues.

The realm of research and development holds immense potential. The field can evolve significantly by directing funds and support towards research dedicated to deepening the understanding of TMJ disorders and exploring innovative treatment modalities. Future research should also critically assess the effectiveness of various treatments, including those beyond the scope of conventional dental care.

An often overlooked but critical aspect of TMJ management is patient education. Dentists have a responsibility to demystify TMJ disorders for their patients, offering clear and accessible information and empowering them with strategies to manage their TMJ condition at home and to address dental fear associated with TMJ conditions [52]. This encompasses exercises for the jaw, stress reduction techniques, and advice on avoiding behaviors that exacerbate the disorder [53].

In today’s technologically driven world, integrating digital tools and techniques in diagnosing and treating TMJ disorders is a natural progression. From advanced imaging technologies for precise diagnosis to the use of computer-aided design (CAD) in creating occlusal splints, technology can play a transformative role. Additionally, therapies such as biofeedback might be considered, particularly for stress-related TMJ issues.

Underpinning all of these efforts are robust quality assurance measures. Such measures are crucial in monitoring the outcomes of treatments and gauging patient satisfaction, ensuring that TMJ disorders adhere to the highest standards and reflect current evidence-based practices. Additionally, integrated training and education involving regular interdisciplinary workshops or seminars can foster an exchange of knowledge and case studies pertinent to TMD treatment [54]. Streamlined communication channels, such as regular case meetings and shared digital platforms, can enhance the efficiency of patient care.

Coordinated patient management is crucial, involving joint assessments and the development of collaborative treatment plans. Engaging in collaborative research projects and sharing findings through medical journals will contribute to the body of knowledge on TMD treatment. Patient engagement through joint workshops and educational materials covering both dental and physiotherapy aspects of TMD is essential [55]. Regular patient and professional feedback loops can help refine collaborative treatment approaches.

To improve the management of Temporomandibular Joint Disorders (TMDs) in dental practice, it is essential to enhance both initial and continuing dental education by including comprehensive modules on TMDs in undergraduate and postgraduate programs. This would equip practitioners with a deeper understanding and more effective management strategies. Interprofessional training programs that involve dentists, physiotherapists, and other relevant healthcare professionals should also be established. These programs would facilitate better understanding and teamwork among different specialists, leading to more holistic and patient-centered care for TMDs. There is a dire need to develop and adopt integrated treatment protocols that combine dental and physical therapy interventions for a more comprehensive approach to TMD management, potentially leading to better patient outcomes.

Raising awareness among dental practitioners about the benefits and roles of physical therapy in TMD treatment is crucial. This can be achieved through workshops, seminars, and inclusion in continuous professional development activities. Creating platforms and opportunities for improved communication and collaboration between dentists and physiotherapists is also important. This could include joint conferences, collaborative research projects, and shared patient care teams. Establishing clear guidelines and protocols for referring TMD patients to physiotherapists and other specialists would help standardize care and ensure that patients receive the most appropriate treatment.

Supporting research initiatives focused on TMD enhances the understanding of these disorders and develops innovative treatment methods. Encouraging participation in research can help practitioners stay abreast of the latest trends and best practices in TMD management. Developing patient education materials and programs is also vital. This could include information on exercises, stress management techniques, and other lifestyle modifications that can alleviate TMD symptoms. Utilizing modern technology, such as digital diagnostic tools and treatment planning software, can improve the accuracy and efficacy of TMD diagnosis and treatment.

Finally, implementing quality assurance measures to monitor the effectiveness of TMD treatments and gathering patient feedback are essential steps in continuously improving care delivery. By incorporating these approaches, dental practitioners can significantly enhance the quality of care provided to patients with TMD, leading to improved health outcomes and greater patient satisfaction.

## Conclusion

Our findings confirm that the current questionnaire is both reliable and valid, making it a valuable tool for assessing dentist knowledge regarding the importance of physical therapy in treating patients with TMDs. According to our findings, dental practitioners encounter patients with temporomandibular joint disorders (TMDs) tend to employ individualized approaches in their practice. Moreover, our study highlights significant gaps in both knowledge and practical application within this cohort. While the dentist’s firsthand accounts provide valuable insights into the clinical realities of managing TMJ disorders within a dental setting.

Dental practitioners face many challenges when treating TMD because of their heterogeneous nature, variability in treatment responses, and the limitations of conventional therapeutic approaches. As a result, there is a need for more integrative and interdisciplinary approaches in treating TMJ disorders, especially given that the dentist admits to having limited knowledge and does not collaborate with other healthcare professionals, such as physiotherapists. In our study, we highlight how educational attainment shapes practitioners’ approach to TMD management and highlight the importance of enhancing interdisciplinary collaboration.

Our study indicates clearly that there is a need for enhanced CPD opportunities on TMJ disorders and for dental education to be approached more holistic. To effectively and collaboratively, dental professionals must address these gaps. A more holistic and interdisciplinary approach to health care will be fostered through improvements in educational and professional development programs.

While the dentist’s focus on avoiding interference of TMJ disorders with dental treatments is understandable, it also highlights the need for more integrated care approaches. Practitioners can improve both the management of TMJ disorders and the overall quality of dental care by acknowledging the complexities and interconnections between TMJ disorders and general dental health. Although the study has limitations, it serves as a crucial discussion starter, raising awareness of the complexities involved in the diagnosis, treatment, and management of TMJ disorders in dentistry. As a result, the paper emphasizes the importance of evidence-based practice, ongoing education, and multidisciplinary approaches.

Finally, this study emphasizes the need for broader research incorporating diverse dental practitioners’ perspectives and patient experiences to understand the management of TMJ disorders. Understanding such research would lead to better clinical practices, improved patient outcomes, and more effective dental professional development programs.

## Data Availability

No datasets were generated or analysed during the current study.

## Abbreviations

TMDs: Temporomandibular Joint Disorders
TMJ: Temporomandibular Joint
DC/TMD: Diagnostic Criteria for Temporomandibular Disorders
PTs: Physical Therapists
PT: Physical Therapy
CI: Confidence Interval
ADA: American Dental Association
CPD: Continuing Professional Development
